# Effect of Formulation Variables on the Stability of a Live, Rotavirus (RV3-BB) Vaccine Candidate using *in vitro* Gastric Digestion Models to Mimic Oral Delivery

**DOI:** 10.1016/j.xphs.2020.09.047

**Published:** 2021-02

**Authors:** Prashant Kumar, Swathi R. Pullagurla, Ashaben Patel, Ravi S. Shukla, Christopher Bird, Ozan S. Kumru, Ahd Hamidi, Femke Hoeksema, Christopher Yallop, Julie E. Bines, Sangeeta B. Joshi, David B. Volkin

**Affiliations:** a Department of Pharmaceutical Chemistry, Vaccine Analytics and Formulation Center, University of Kansas, Lawrence, KS, 66047, USA; b Batavia Biosciences B.V., Bioscience Park Leiden, Zernikedreef 16, 2333 CL Leiden, the Netherlands; c Murdoch Children's Research Institute, Department of Paediatrics University of Melbourne, Department of Gastroenterology and Clinical Nutrition, Royal Children's Hospital, Parkville, Victoria, Australia 3052

**Keywords:** Rotavirus, RV3-BB, Live virus vaccine, Formulation, Stability, Oral delivery

## Abstract

In this work, two different *in vitro* gastric digestion models were used to evaluate the stability of a live attenuated rotavirus vaccine candidate (RV3-BB) under conditions designed to mimic oral delivery in infants. First, a forced-degradation model was established at low pH to assess the buffering capacity of formulation excipients and to screen for RV3-BB stabilizers. Second, a sequential-addition model was implemented to examine RV3-BB stability under conditions more representative of oral administration to infants. RV3-BB rapidly inactivated at < pH 5.0 (37 °C, 1 h) as measured by an infectivity RT-qPCR assay. Pre-neutralization with varying volumes of infant formula (Enfamil®) or antacid (Mylanta®) conferred partial to full protection of RV3-BB. Excipients with sufficient buffering capacity to minimize acidic pH inactivation of RV3-BB were identified (e.g., succinate, acetate, adipate), however, they concomitantly destabilized RV3-BB in accelerated storage stability studies. Both effects were concentration dependent, thus excipient optimization was required to design candidate RV3-BB formulations which minimize acid-induced viral inactivation during oral delivery while not destabilizing the vaccine during long-term 2–8 °C storage. Finally, a statistical Design -of-Experiments (DOE) study examining RV3-BB stability in the *in vitro* sequential-addition model identified key formulation parameters likely affecting RV3-BB stability during *in vivo* oral delivery.

## Highlights

• Formulation development for oral delivery of a live, rotavirus vaccine candidate (RV3-BB) is described.
• RV3-BB virus stability during *in vivo* oral delivery was mimicked with two different *in vitro* gastric digestion models.
• Pre-neutralization with infant formula (Enfamil®) or antacid (Mylanta®) conferred partial to full protection of RV3-BB.
• Formulation excipients (succinate, acetate, adipate) minimized acidic pH inactivation of RV3-BB without preneutralization.

## Introduction

Rotaviral infection remains a leading cause worldwide of severe diarrhea in children <5 years of age, despite the availability of protective vaccines.^1^ The past 15 years have witnessed remarkable progress in rotavirus (RV) vaccination programs starting with regulatory approval of two live, orally administered viral vaccines, RotaTeq® and Rotarix®.^2^, ^3^, ^4^, ^5^ More recently, two additional, orally administered live RV vaccines have received WHO prequalification approval including Rotavac® and ROTASIIL®.^3^^,^^6^^,^^7^ Nevertheless, global coverage is currently only 48% worldwide, leading to an estimated 215,000 deaths annually mostly in the low and middle income countries (LMICs), due to high vaccine cost, limited manufacturing capacity, and lower vaccine efficacy in low-resource settings.^8^, ^9^, ^10^ Considering these challenges, the ongoing RV3-BB vaccine development program aims to further strengthen current RV vaccination efforts by developing a low-cost, orally delivered live RV vaccine that not only targets RV strains more prevalent in LMICs, but could also offer protection in neonates.^8^^,^^11^

The RV3-BB virus is a live, asymptomatic, naturally attenuated, human monovalent RV developed at Murdoch Children's Research Institute (MCRI) from human neonatal RV strain RV3 (G3P[6]). RV3-BB is adapted for replicating in newborn gut even in the presence of maternal antibodies and when the baby is breast-fed. These intrinsic features of RV3-BB makes it suitable for providing early protection to infants at birth while minimizing safety concerns due to intussusception.^8^^,^^12^^,^^13^ Early and mid-stage RV3-BB clinical trials are ongoing in adults, infants and neonates to assess safety and efficacy in different clinical settings,^8^^,^^14^, ^15^, ^16^, ^17^, ^18^, ^19^ using a frozen liquid formulation combined with pretreatment regimens to neutralize gastric acid (including the use of Mylanta®). Commercially available liquid RV vaccines, however, are stored at 2–8 °C, and formulated to eliminate the need for preneutralization of gastric acid prior to administration.^3^ Thus, in order to match this target product profile as well as to lower costs, RV3-BB vaccine candidate underwent formulation development and promising results have recently been described (Kumar, Shukla et al., manuscript submitted).^20^

During the RV3-BB formulation development program, it became evident that a key challenge was excipient effects on virus stability within the primary container during long-term storage at 2–8 °C vs. virus stability in a low-pH, 37 °C environment encountered in the stomach during oral delivery. Similar challenges have been described with the development of the RotaTeq® vaccine including the use of a clinical trial to compare different formulations with and without preneutralization.^21^ Since the ability to assess different formulation variables is greatly limited if clinical trials are required, alternative *in vitro* approaches are needed to prescreen candidate formulations prior to any clinical assessments. Examples of *in vitro* digestion models to mimic infant stomach conditions include examining food digestion^22^ and antacids, with various versions of the latter having been used to determine the acid neutralizing capacity (ANC) of various excipients during formulation development of Rotarix® and Rotavac®.^4^^,^^7^

In this work, we describe two different *in vitro* digestion models to evaluate RV3-BB stability vs. various formulation parameters under conditions that mimic *in vivo* oral administration. First, a forced degradation model (i.e., low pH, 37 °C) was used to not only evaluate the buffering capacity of various formulations, but to test their ability to protect the *in vitro* potency of RV3-BB as measured by infectivity RT-qPCR assays. Second, a sequential-addition *in vitro* model was used to assess RV3-BB stability under conditions more realistic to mimic infant stomach including varying HCl levels, addition of pepsin, and the presence of infant formula. Finally, we further evaluated key formulation variables using a statistical Design of Experiments (DOE) approach to examine RV3-BB stability under conditions that mimic oral delivery to support selection of candidate RV3-BB vaccine formulations for future clinical studies.

## Materials and Methods

### Materials

Biological materials used during this study were secured by Batavia Biosciences, the Netherlands, as part of collaboration agreements with MCRI and PT-BioFarma: the RV3-BB seed was from MCRI, the Bulk Drug Substance was from PT-BioFarma, the MA104 cells and the RV3-BB reference standard were obtained from Batavia Biosciences (produced at Batavia using the RV3-BB virus stock from PT-BioFarma).

Sucrose, disodium phosphate and sodium dihydrogen phosphate were purchased from EMD-Millipore, USA. All other excipients were obtained from Sigma-Aldrich, USA except for sodium acetate from Fluka, USA. A solution of 37% (w/v) HCl was purchased from Acros Organics. Pepsin and sodium succinate were purchased from Sigma-Aldrich, USA. TaqMan® Fast Virus 1-Step Master Mix was purchased from Applied Biosystems (ThermoFisher, USA). Enfamil® (soy infant formula) was obtained from Mead Johnson and Mylanta® (maximum strength) was procured from McNeil Consumer Pharmaceutical Co., USA.

### Methods

#### Virus Quantification

RV3-BB *in vitro* potency quantification was performed using quantitative Reverse-Transcription Polymerase Chain Reaction (RT-qPCR) assay as described in detail elsewhere (Kumar, Shukla et al., manuscript submitted).^20^ Briefly, MA104 cells were plated in 96-well plates and infected with serial dilutions of RV3-BB standard and 50-fold dilution of the test samples. After 18 ± 0.5 h incubation at 37 °C, Triton X-100 was added and the cells were lysed by freeze-thaw. VP7 gene-specific primers and probe were used for quantification of the mRNA produced during RV3-BB replication using Taq polymerase in QuantStudio® 7 Flex Real-Time PCR System (Applied Biosystems, USA). RV3-BB log loss was calculated by subtracting virus log titer in test formulations (1 h at 37 °C, with HCl) from that in control formulations (incubated at 4 °C with no HCl). Propagation of error for log loss of virus titer (vs. unstressed control) were calculated using equation SE(C) = √(SE(A)ˆ2 + SE(B)ˆ2).

#### Preparation of Virus Liquid Formulation

Concentrated excipient stock solutions were prepared, pH adjusted to 7.8 and sterile filtered using a 0.22 μm PVDF filter (Millipore). Calculated amounts of the excipient stocks were combined with media, which was then mixed with RV3-BB bulk drug substance in 50 mL sterile conical tubes. All formulation preparations were carried out in a class II biosafety cabinet (Labconco, USA). The two main formulations used in this study contained 60% (w/v) sucrose, 0.01% (w/v) PEG 3350, 25% (v/v) Dulbecco's Modified Eagle Medium (DMEM) in a sodium phosphate buffer at pH 7.8. Formulation A also contained 400 mM sodium succinate while formulation B does not contain sodium succinate (unless otherwise stated).

#### pH Stability Profile of RV3-BB Virus

RV3-BB bulk drug substance was diluted in 100 mM succinate phosphate buffer across a pH range of 3–6 (at 0.5 pH unit increments) both in presence and absence of 2000 U/mL pepsin. The viral samples were incubated at 37 °C for 1 h (physiological temperature and average residence time in the infant stomach)^22^ for studying virus potency loss with respect to pH. At the end of 1 h incubation, virus samples were immediately neutralized by 50 fold dilution using DMEM (supplemented with 1 μg/mL porcine trypsin) and *in vitro* potency was determined by infectivity RT-qPCR assay. Viral potency losses are described as log loss (titer loss against a specific stress condition) vs unstressed control (neutral pH, 4 °C control) prepared in the same succinate phosphate buffer.

#### Accelerated Storage Stability of RV3-BB Virus

Accelerated storage stability studies were carried out at 25 °C for 1-week vs unstressed control samples stored at −80 °C for same time. The formulations for this study were prepared at 0, 50, and 200 mM excipient concentrations (i.e., sodium acetate, sodium succinate, malic acid, adipic acid, or citric acid; referred to as acetate, succinate, malate, adipate and citrate, respectively, for simplicity) in presence and absence of 30% (w/v) sucrose, pH 7.0 (suboptimal sucrose concentrations and solution pH were used to further accelerate these storage conditions). Both thermal stressed and unstressed samples were stored at −80 °C until RT-qPCR analysis, and hence were each subjected to 1 freeze-thaw cycle.

#### Forced Degradation *In Vitro* Digestion Model

**Fig. 1:**
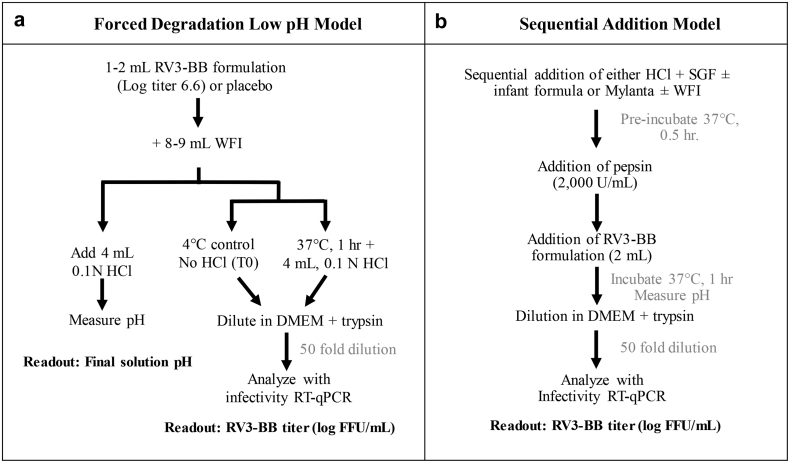
Experimental outline of the two *in vitro* digestion models used to examine RV3-BB stability under conditions to mimic *in vivo* oral delivery. (a) Forced degradation, low pH model used to assess buffering capacity of various excipients and to screen for RV3-BB stabilizers, and (b) sequential-addition model to evaluate RV3-BB stability under conditions that better mimic oral administration in infants. RV3-BB stability was determined by RT-qPCR infectivity assays as described in text. SGF - simulated gastric fluid, WFI - water for injection, DMEM - Dulbecco's Modified Eagle Medium.

The stability of RV3-BB viral titers in the forced degradation *in vitro* model was evaluated under conditions adapted from elsewhere^4^ and as outlined in Fig. 1a. First, the buffering capacity of various buffers and excipients was determined by addition of 8 mL water for injection (WFI) to a 2 mL placebo RV3-BB candidate formulation at 37 °C. Then, 4 mL 0.1 N HCl was added and pH was recorded before and after acid addition. The final reaction volume of 14 mL is representative of an infant stomach.^4^ By measuring the pH change under each experimental condition, we can rank order the buffering capacity of each formulation in this study, albeit we are not directly determining/calculating the actual buffering capacity value. Excipient combinations providing final pH values consistent with those obtained with 2 mL Mylanta® were further evaluated for their ability to stabilize RV3-BB viral titers in this model. For this, 4 mL 0.1 N HCl was added to 10 mL of RV3-BB formulations, diluted with Water for Injection (WFI) and incubated at 37 °C for 1 h to observe log loss of RV3-BB titer vs. unstressed control (without HCl at 4 °C). After incubation, all RV3-BB containing samples were immediately diluted 50-fold with DMEM (supplemented with 1 μg/mL porcine trypsin) to neutralize the acidic pH prior to RT-qPCR analysis.

#### Sequential-addition *In Vitro* Digestion Model

The stability of RV3-BB viral titers in the sequential-addition *in vitro* model was evaluated under conditions adapted from elsewhere^22^ and as outlined in Fig. 1b. The model was simplified by omitting oral and intestinal phases^22^ and scaled-down in volume to conserve RV3-BB viral bulks. The stability of RV3-BB viral titers in various formulations was examined under the following conditions: meal (infant formula Enfamil®) to simulated gastric fluid (94 mM sodium chloride and 13 mM potassium chloride, pH 5.3) volume ratio of 63:37, final pepsin concentration of 2000 U/mL, and 0.1 N HCl addition in the range of 0–4 mL (based on Geigy Scientific Table^23^) with concomitant addition of WFI to maintain constant volume. Pepsin activity was verified using standard pepsin assay. The *in vitro* model included addition of pepsin and RV3-BB formulation to a mixture of HCl, simulated gastric fluid, and meal or Mylanta® (preincubated at 37 °C for 30 min to achieve the set temperature), and then incubated at 37 °C for 1 h. The loss in viral titer was determined by log titer loss (vs. unstressed control sample without HCl at 4 °C). The final reaction volume of 14 mL used in this study is representative of infant stomach.^4^ RV3-BB samples were then diluted 50-fold with DMEM (supplemented with 1 μg/mL porcine trypsin) to neutralize the acidic pH prior to RT-qPCR analysis. Studies evaluated the effect of varying HCl levels (in a range of 0–4 mL 0.1 N HCl), excipient concentration in the formulation (0–400 mM succinate), presence of pepsin (2000 U/mL), the use of meal (5–8 mL Enfamil®) or Mylanta® (0–2 mL). Equal volumes of WFI was substituted for meal and HCl in experiments to bring total volume to 14 mL.

#### Design of Experiments Study

A statistical DOE approach involving three level response Surface Methodology Central Composite Design, RSM-CCD using JMP 14.1.0 software (SAS Institute Inc., USA) was performed. The DOE study evaluated the inter-relationships between key input variables and their ranges (lower and upper values) including: (1) 0.1 N HCl addition (0.5, 4.0 mL), (2) succinate concentration in candidate formulations (50 mM, 400 mM), and (3) Enfamil® pre-feeding (5 mL, 8 mL). The output was measured in terms of stability of RV3-BB infectivity titers (log loss) and final solution pH using the modified sequential-addition *in vitro* model. Model fitting and prediction of formulation variables was performed using prediction profiler feature of the JMP 14.1.0 software.

## Results

In this work, two different *in vitro* gastric digestion models were used as part of formulation development of a candidate RV3-BB vaccine as described in Fig. 1. The first model is a forced-degradation, low pH setup at 37 °C used to determine buffering capacity of various excipients and then to screen for the effect of additives on RV3-BB stability (Fig. 1a). This model is a modified version of the Baby Rossette Rice Assay.^4^ The second model is a sequential-addition setup at 37 °C under conditions that more realistically mimic conditions encountered during oral administration of vaccine to infants (Fig. 1b). This second model is based on *in vitro* models developed for studying food digestion.^24^, ^25^, ^26^, ^27^ In particular, we adapted a static model that Menard et al. recently proposed to mimic infant digestion,^22^ including a ~4X scaled-down version to use less RV3-BB viral bulks for these studies.

**Fig. 2:**
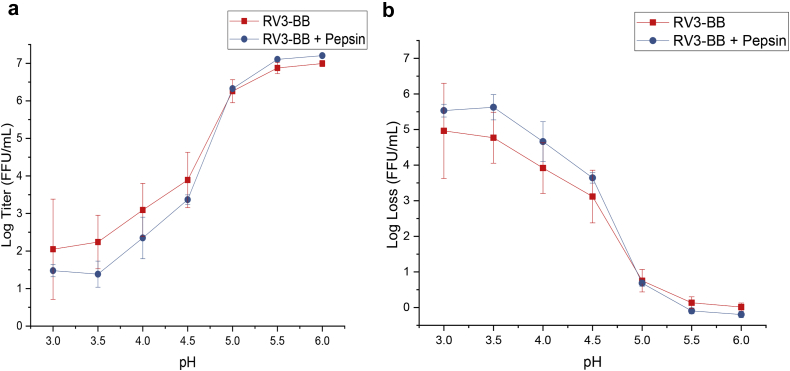
pH stability profile of RV3-BB as measured by infectivity RT-qPCR, (a) RV3-BB viral titer values (Log (FFU/mL)) and (b) RV3-BB titer losses (log loss vs. unstressed control). Samples were incubated for 1 h, 37 °C in the presence and absence of 2000 U/mL pepsin in 100 mM succinate-phosphate buffer (from pH 3.0 to 6.0). RV3-BB samples were prepared at a titer of 6.6 (Log (FFU/mL)) and stability data are presented as the mean ± SD (n = 4).

Prior to evaluating RV3-BB stability in these two *in vitro* gastric digestion models, the pH stability profile of RV3-BB across a pH range of 3.0–6.0 was established (Fig. 2). Virus samples were examined at 0.5 pH unit increments, incubated at 37 °C for 1 h in the presence or absence of pepsin, and virus potency loss was determined by infectivity RT-qPCR assay. The results show that RV3-BB is stable above pH 5.5 with no notable change in virus titer (Fig. 2a) or virus titer log loss vs. control (Fig. 2b). A sharp decline in RV3-BB titer, however, was observed as a function of decreasing solution pH range (pH 3.0–5.0) that clearly demonstrated RV3-BB is labile under more acidic conditions. No notable effect of pepsin at 2000 U/mL was observed on RV3-BB *in vitro* potency under these conditions.

### Forced Degradation, Low pH *In Vitro* Digestion Model

We first examined pretreatments to neutralize gastric acid previously used in RV3-BB clinical trials, namely 2 mL of the antacid Mylanta®^8^^,^^17^). After addition of 2 mL of Mylanta® to the forced degradation, low pH *in vitro* model (Fig. 1a), pH values of 5.3 was observed after addition of 4 mL of 0.1 N HCl. Interestingly, 2 mL Mylanta® maintains the solution pH above 5.0, which is consistent with pH stability profile of RV3-BB virus (Fig. 2). Since 2 mL of Mylanta® has been successfully used in RV3-BB clinical trials to pre-neutralize gastric acid, this pH value was then used as a benchmark to evaluate candidate RV3-BB formulations. The candidate RV3-BB formulations examined in this model contained various ANC excipients (to neutralize gastric acid upon oral delivery) as well as phosphate or histidine buffers (to maintain solution pH and stabilize RV3-BB during long-term storage as described elsewhere, Kumar, Shukla et al., manuscript submitted).^20^ As shown in Table 1, candidate RV3-BB formulations have been grouped by different colors based on the pH value obtained by the addition of 2 mL Mylanta® (i.e., pH 5.3). The green color represents final pH values of ≥5.3 (similar to the final pH using 2 mL of Mylanta®), the orange color represents final pH values in a range of 4.6–5.3 (approximate to the results with 2 mL Mylanta®), and the red color with final pH < 4.6 (much lower pH compared to using 2 mL Mylanta®). The ANC excipients formulated at higher concentrations and at a higher initial pH values more effectively resisted pH changes after addition of 4 mL 0.1 N HCl. As expected, the ANC excipients best resisted pH shifts near their pKa values (Table 1).

**Table 1:** Buffering Capacity Assessment of Various Excipients in Three Different Buffers as a Function of Their Potential Concentration Ranges in Candidate RV3-BB Formulations as Determined in the Forced Degradation, Low pH Model. Solution pH Values Were Determined After Addition of 4 mL 0.1 N HCl at 37 °C. Solution pH Values After Addition of Mylanta® in this Model was 5.3 and the Color Code Below are Relative to the Mylanta® Results. See Fig. 1a for Experimental Details.

N/A- not available; The pKa values of additives are as follows: acetic acid (4.8), citric acid (3.1, 4.8, 6.4), succinic acid (4.2, 5.6), malic acid (3.4, 5.2), maleic acid (1.9, 6.1), Tris (8.1), HEPES (3.0, 7.5), carbonic acid (6.4, 10.3), adipic acid (4.4, 5.4).

**Fig. 3:**
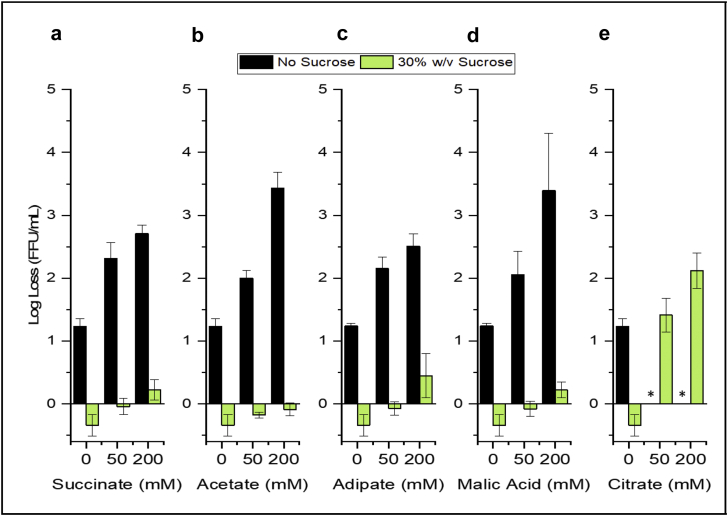
Effect of five different ANC excipients on accelerated storage stability showing RV3-BB titer losses in different formulations as measured by infectivity RT-qPCR assays. RV3-BB *in vitro* potency losses after 1 week at 25 °C (vs unstressed control samples stored at −80 °C) were evaluated both in the presence and absence of 30% (w/v) sucrose in a phosphate buffer, pH 7.0 prepared at a titer of 6.6 (Log (FFU/mL)). The effect of the addition of five excipients on RV3-BB storage stability was determined at indicated concentrations: (a) Succinate, (b) Acetate, (c) Adipate, (d) Malic Acid, and (e) Citrate. ∗ Citrate resulted in the complete loss of RV3-BB titer in the no sucrose control at −80 °C. The data are presented as the mean ± SD (n = 3).

Based on these results, five additives (sodium acetate, sodium succinate, malic acid, adipic acid, citric acid) were identified as promising, and were further evaluated at varying concentrations (0, 50 and 200 mM) for their effect on RV3-BB viral titers during an accelerated stability study (25 °C, 1 week) in a phosphate buffer at pH 7.0 in presence and absence of 30% (w/v) sucrose (sucrose stabilizes RV3-BB as described elsewhere, Kumar, Shukla et al., manuscript submitted^20^). In the presence of 30% (w/v) sucrose, four additives (sodium acetate, sodium succinate, malic acid and adipic acid) showed minimal RV3-BB titer losses (log loss vs unstressed −80 °C control) of <0.5 (Fig. 3a–d). In contrast, 2–4 log loss of RV3-BB titer was observed in the absence of sucrose with the same four additives. Citric acid (50 and 200 mM) was eliminated for future use due to its detrimental effect on RV3-BB stability. In the presence of 30% (w/v) sucrose, 1–2 log loss of RV3-BB titer was observed with citrate (Fig. 3e), and in the absence of sucrose, citrate addition led to viral titer losses in the unstressed −80 °C control as well. At the same time, we have shown that these additives (succinate, acetate and adipate) preserve RV3-BB log titers by resisting pH changes upon acid challenge (4 mL of 0.1 N HCl and 1 h incubation at 37 °C) in a concentration dependent manner (0–500 mM), with higher additive concentration being more effective in maintaining RV3-BB virus potency and stability (Kumar, Shukla et al., manuscript submitted).^20^

### Sequential-addition *In Vitro* Digestion Model

To evaluate the stability profile of RV3-BB under conditions that better mimic oral administration under infant gastric conditions, a sequential-addition *in vitro* digestion model was employed as described above in Fig. 1b. With this setup, the stability of RV3-BB could be examined at varying levels of HCl addition (and thus varying final solution pH) as well as in the presence of pepsin. Key formulation parameters such as different concentrations of ANC excipients in candidate formulations, as well as the effect of administration variables such as the use of infant formula (i.e., pre-feeding or meal effects) on RV3-BB stability were examined and compared to Mylanta® administration (as a control) as described below.

**Fig. 4:**
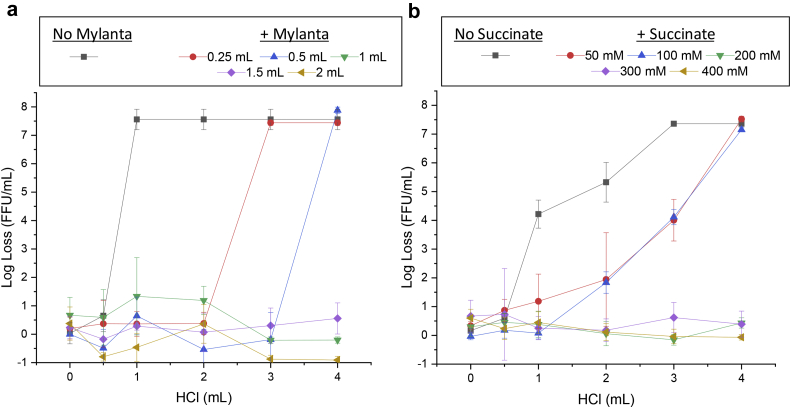
Effect of Mylanta® and succinate on the stability of RV3-BB in the sequential-addition *in vitro* digestion model as measured by infectivity RT-qPCR, (a) Mylanta® (0–2 mL) and (b) succinate (0–400 mM). RV3-BB infectivity titer losses (log loss vs. unstressed control without HCl) after 1 h, 37 °C are presented as the mean ± SD (n = 4). RV3-BB formulation (60% (w/v) sucrose, 0.01% (w/v) PEG, 25% (v/v) DMEM, in a phosphate buffer at pH 7.8) was used in case of Mylanta® addition, while same formulation with 0–400 mM succinate was used in the case of succinate addition. All experiments were performed in absence of infant formula (Enfamil®) and prepared at a titer of 6.6 (Log (FFU/mL).

First, we titrated addition of 0–2 mL of Mylanta® in terms of RV3-BB stability in this model. The results show 0.25–0.5 mL Mylanta® provided only partial protection while 1.0, 1.5 and 2.0 mL Mylanta® essentially completely protected RV3-BB potency as measured by infectivity RT-qPCR assays (Fig. 4a). One of the promising ANC additives succinate, which also showed good RV3-BB storage stability in candidate formulations (Kumar, Shukla et al., manuscript submitted),^20^ was then evaluated in this model. As displayed in Fig. 4b, 50 and 100 mM succinate only partially protected RV3-BB viral titers (compared to absence of succinate control), while 200 and 400 mM succinate completely protected RV3-BB viral titer values under these conditions.

**Fig. 5:**
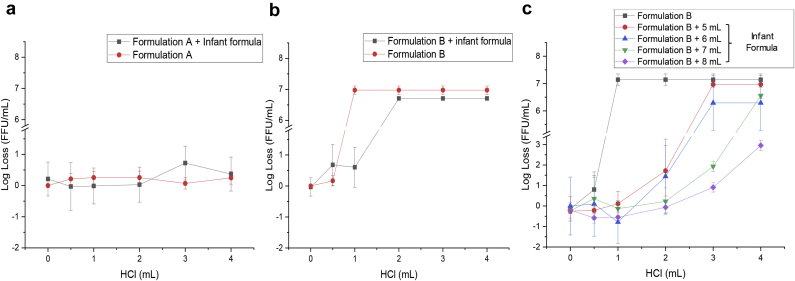
Effect of infant formula (Enfamil®) addition on RV3-BB stability in the sequential-addition *in vitro* digestion model as measured by infectivity RT-qPCR, (a) candidate formulation A (containing 400 mM succinate), (b) candidate formulation B (same as formulation A but without succinate). RV3-BB infectivity titer losses (log loss vs. unstressed control without HCl) after 1 h, 37 °C are presented as the mean ± SD (n = 4). Formulations contained a titer of 6.6 (Log (FFU/mL)) with 60% (w/v) sucrose, 0.01% (w/v) PEG, 25% (v/v) DMEM, in a phosphate buffer pH 7.8, either in the presence of 400 mM succinate (Formulation A) or without succinate (Formulation B).

To examine meal effects on RV3-BB viral titers in this model, the effect of 400 mM succinate in a candidate formulation (see Fig. 5 legend for composition) in presence and absence of 5 mL Enfamil® was examined. In the presence of 400 mM succinate (Formulation A), essentially no RV3-BB titer loss was observed, in the presence or absence of 5 mL Enfamil®. For the same formulation without succinate (Formulation B), a small protective effect was noted with complete loss in virus titer after addition of 1.0 mL of HCl in the absence of a meal, and 2 mL of HCl in the presence of 5 mL Enfamil®. Finally, the effect of titrating HCl in a RV3-BB formulation without succinate (Formulation B) in the presence of 5–8 mL Enfamil® was performed to evaluate the effect of increasing meal volume on protecting RV3-BB viral titers. Although a complete viral titer loss of RV3-BB was observed upon the addition of 1 mL 0.1 N HCl in absence of meal, notable partial protection as a function of increasing meal volume was observed (Fig. 5c). For example, no loss in RV3-BB titers were seen at 1 mL 0.1 N HCl, and partial losses were observed at 2 and 3 mL of acid addition.

### Design of Experiments (DOE) Studies

The results described above utilized a one-factor-at-a-time experimental design (e.g., varying the levels of either HCl, infant formula, succinate) to evaluate their effects on RV3-BB stability and solution pH. Such studies, however, cannot address interactions that may occur between these variables. We therefore employed a statistical DOE approach with the sequential-addition *in vitro* model to better understand potential inter-relationships between three input variables including (1) amount of 0.1 N HCl added, (2) succinate concentration in candidate formulations, and (3) use of infant formula as pre-feeding. The two outputs that were experimentally determined included RV3-BB viral titer stability and solution pH values. The experimental design included 16 runs of various combinations of input variables as displayed in Table 2.

**Table 2:** Design of Experiments (RSM-CCD) Experimental Matrix for Assessing RV3-BB Viral Titer Stability and Final Solution pH in the Sequential-addition *in vitro* Digestion Model. Experimental (input) Variables Included Amount of HCl Added in the Model, Concentration of Succinate in Candidate Formulations, and Amount of Pre-feeding (Enfamil® addition). The Experimentally Measured Values and Predicted Values (from DOE model) for RV3-BB Potency Log Losses and Final Solution pH are Shown, Respectively.

Run No.	Input Variables			RV3-BB Titer Log Loss		Final Solution pH	
	HCl (mL)	Succinate (mM)	Enfamil® (mL)	Measured	Predicted	Measured	Predicted
1	4	50	8	0.8	1.0	5.0	4.9
2	0.5	400	8	0.4	0.8	6.8	6.7
3	2.25	400	6.5	0.4	0.2	6.0	6.0
4	4	50	5	3.4	2.9	4.4	4.4
5	0.5	225	6.5	0.4	0.2	6.8	6.9
6	0.5	50	5	0.8	0.9	6.6	6.6
7	0.5	50	8	0.2	0.1	6.7	6.7
8	2.25	225	8	0.4	0.1	5.9	6.0
9	2.25	225	5	0.4	0.7	5.9	5.8
10	4	225	6.5	0.8	1.1	5.4	5.3
11	2.25	225	6.5	0.5	0.4	5.9	5.9
12	2.25	50	6.5	0.6	0.9	5.5	5.5
13	4	400	5	0.7	0.8	5.6	5.6
14	0.5	400	5	0.2	0.0	6.9	6.9
15	2.25	225	6.5	0.4	0.4	5.8	5.9
16	4	400	8	0.7	0.5	5.7	5.8

**Fig. 6:**
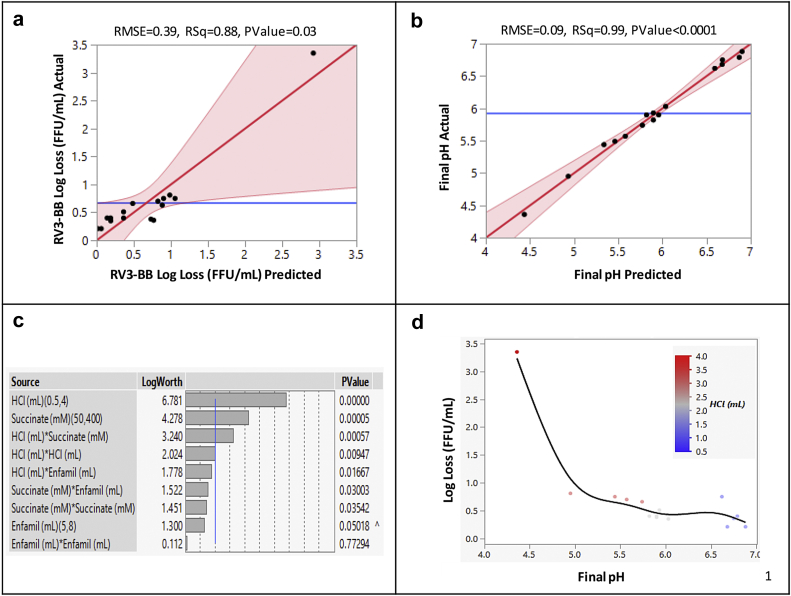
Parameters of Design of Experiments model (RSM-CCD model) used to assess stability of RV3-BB infectivity titers and final solution pH in the sequential-addition *in vitro* digestion model. Parity plots showing statistically significant (p ≤ 0.05) correlation between experimentally measured values vs. DOE model predicted values for (a) RV3-BB stability (log loss) as measured by infectivity RT-qPCR, and (b) final solution pH. Panel (c) shows significant experimental factors affecting RV3-BB potency losses and final pH responses. (d) Experimentally measured RV3-BB *in vitro* potency log losses vs. final solution pH from RSM-CCD matrix showing rapid RV3-BB log titer loss below pH 5.0.

The experimental results for RV3-BB viral titer stability and solution pH, along with the predicted results based on the DOE modeling, are summarized in Table 2. Additional data analysis results from the DOE are shown in Fig. 6. Statistically significant fits were demonstrated between the experimental and predicted values with a p-value of <0.03 for RV3-BB titer losses (Fig. 6a) and a p-value of <0.0001 for final solution pH (Fig. 6b). Both the succinate concentration in formulation and the amount of HCl added were identified as the key experimental factors significantly affecting the measured responses (Fig. 6c). Finally, from the DOE modeling, a rapid decrease in RV3-BB titer was predicted when the final solution pH fell below pH 5.0 which closely matched the experimental data (Fig. 6d). This result is also consistent with the experimentally determined pH stability profile of RV3-BB (Fig. 2).

Using the experimental data and the DOE mathematical modeling, a high correlation between final solution pH and RV3-BB titer losses can be used to predict the succinate concentration needed in candidate formulations to protect RV3-BB under conditions that mimic the infant stomach (with and without pre-feeding). Based on published values of acid production shown in Table 3, the DOE model predicts that with exposure to 4 mL 0.1 N HCl addition, a candidate RV3-BB formulation that contains 220–350 mM succinate, in the presence of 5–8 mL infant formula, provide sufficient buffering capacity to keep the final solution pH > 5.0. With the addition of less 0.1 N HCl such as 3 mL or 1.5–1.9 mL, 110–215 mM succinate or 50 mM succinate, respectively, in the candidate RV3-BB formulations would be required.

**Table 3:** Predicted Concentration of Succinate Required in Candidate RV3-BB Formulations to Provide Sufficient Buffering Capacity for Oral Delivery to Infants Using DOE Analysis of the Experimental Results from the Sequential-addition *in vitro* Digestion Model. The Predicted Values Were Based on the Assumption of Maintaining the Final Solution pH at Lower Bound of pH 5.3. The Succinate Levels Were Predicted Using Prediction Profiler Feature of the JMP 14.1.0 Software.

Infant Age	HCl		Enfamil® 5.0 mL	Enfamil® 6.5 mL	Enfamil® 8.0 mL
	HCl Production Rate (mmol/h) from Published Literature [1]	HCl (mL) Added to *in vitro* Model to Mimic HCl Production Rate	Predicted Levels of Succinate Required for Oral Delivery (mM)		
9–11 weeks	0.15	1.5	50	50	50
	0.30	3.0	214	146	112
6–7 months	0.19	1.9	50	50	50
	0.40	4.0	348	273	219

## Discussion

Extensive formulation development work has identified promising candidate liquid formulations, stable at 2–8 °C, that can be used for an oral RV3-BB vaccine (Kumar, Shukla et al., manuscript submitted).^20^ Nonetheless, RV3-BB clinical trials will ultimately be needed to establish the final vaccine dose, manufacturing process and formulation. In the case of the commercial RotaTeq® vaccine, the manufacturing process and optimized formulation were clinically evaluated.^28^ Such trials help to establish the “stability window” of the RV vaccine to determine the highest dose that is safe, the lowest dose that is efficacious, and thus, the acceptable potency losses during storage.^29^ It is not possible, however, to rely on clinical trials to evaluate the numerous iterations of manufacturing processes and formulations being considered during development. Thus, this work focuses on addressing *in vitro* approaches to prescreen formulations during development of a RV3-BB live vaccine candidate. In this work, we adapted and utilized two *in vitro* digestion models (based on antacid and food digestion literature) to mimic *in vivo* administration with goal of evaluating the effect of different formulation variables on RV3-BB stability.

We first examined the previous literature to identify *in vitro* models for effectiveness of antacids and food digestion. These approaches vary in complexity ranging from static, semi-dynamic and dynamic models.^24^, ^25^, ^26^, ^27^ For antacids, both the Rossette-Rice and Baby Rossett-Rice titration assays have been described^4^^,^^30^ to examine the harshest conditions found within adult and infant stomachs (e.g., extremes of fasting and HCl concentrations). Such models have been used to determine the acid neutralizing capacity of formulations of commercially available RV vaccines (i.e., Rotarix® and Rotavac®),^4^^,^^7^ yet they have not been used to examine the stability of RV viral titers. For food digestion, both dynamic and semi-dynamic models may provide the most relevant information, however, they are experimentally complex compared to static models which are simpler and cheaper to perform. Recently, an international consensus has been achieved for mimicking adult digestion using a harmonized *in vitro* static model,^22^^,^^31^ yet no such model is available for the infant digestion due to technical, ethical, and financial constraints.^32^^,^^33^ Nonetheless, Menard et al. have recently proposed an *in vitro* static model for infant food digestion^22^ which was adapted in this work to examine the stability of the RV3-BB in candidate formulations.

### RV3-BB Vaccine Stability in Two Different *In Vitro* Digestion Models to Mimic Oral Delivery

RV3-BB clinical studies have used 2 mL Mylanta® for preneutralization of gastric acid prior to oral administration to infants.^8^ Preneutralization of gastric acid during clinical trials using Mylanta® has also been described with other live RV vaccines including G1P1A[8] RV vaccine candidate 89-12 (the precursor to Rotarix®),^34^ and with RotaTeq®.^5^^,^^21^ Based on these considerations, a “compare to Mylanta®” strategy was used in this work to define success criteria of different formulations in terms of final pH and stability of RV3-BB viral titers using the two *in vitro* digestion models. In the forced degradation, low-pH model (Fig. 1a), 2 mL of Mylanta® was required to keep the final pH above pH 5, but in the sequential-addition model (Fig. 1b), 1 mL of the antacid was sufficient. The “compare to Mylanta®” strategy led to the down-selection of promising additives in the forced degradation, low-pH digestion model in terms of (1) maintaining solution pH above pH 5, and (2) minimizing RV3-BB titer losses in the presence of these additives upon acid challenge. These promising additives (sodium acetate, succinate, malic acid, adipic acid, and citric acid), could be ranked-ordered in terms of their protective effect.

At the same time, an accelerated storage stability study (25 °C for 1 week) was performed in the presence of the same down-selected additives and results showed an opposite trend in terms of RV3-BB stability during storage in comparison to the acid challenge study. Among the additives tested, sodium acetate and malic acid had a relatively lower destabilizing effect on RV3-BB titer losses during storage, followed by adipate and succinate, while sodium citrate showed highest titer losses including virus destabilization even in the −80 °C controls (Fig. 3). Divalent cations such as Ca^2+^ have been shown to bind to and stabilize the outer coat proteins VP4 and VP7 of RV,^35^^,^^36^ thus the addition of higher concentrations of these excipients (such as citrate), which have known metal chelating properties, may cause the observed RV3-BB destabilization over time during storage. In contrast, during acid challenge, the pH neutralization effect is the predominant factor to minimize loss of RV3-BB viral titers.

Although RV3-BB displays lability towards sodium citrate, other RV serotypes such as pentavalent, bovine human reassortants G1-G4 and P1[8] in RotaTeq® are formulated in 0.2 M citrate.^5^ A citrate containing buffer is also used for reconstitution of lyophilized human-bovine reassortant pentavalent strains G1-G4 and G9 in ROTASIIL® immediately before oral administration.^3^^,^^4^^,^^7^^,^^37^ In terms of pH stability, other live RV vaccines are formulated at more acidic pH values (e.g., pH 6.2–6.3 for RotaTeq® and Rotarix®) compared to the RV3-BB candidate formulations which showed optimal stability at pH 7.8 (Kumar, Shukla et al., manuscript submitted).^20^ Consistent with this observation, results of this work demonstrate pH lability of RV3-BB below pH 5 in the *in vitro* digestion model, compared to previous reports of the need to maintain the pH above 3.5 for preservation of RV infectivity with RotaTeq®.^5^ In summary, compared to published reports with other RV vaccine strains, RV3-BB appears to be relatively more labile to acidic pH conditions as well as more sensitive to the presence of sodium citrate in the formulation.

The addition of a meal (Enfamil®) provided a partial protective effect on RV3-BB potency when subjected to gastric conditions in the sequential-addition model. For example, increasing the infant formula volume from 5 to 8 mL better protected RV3-BB from potency losses. These results suggest feeding infants before vaccine administration can at least partially protect RV3-BB and thus may reduce the buffering capacity requirements of the formulation. Conversely, allowing a meal prior to RV vaccination may be problematic by inhibiting the infectivity of oral RV vaccines due to the neutralizing activity of milk sIgA antibodies.^38^ Other studies have observed that withholding breastfeeding at the time of RV vaccination did not improve vaccine immunogenicity.^39^^,^^40^ Based on these considerations, both the WHO and CDC do not recommend withholding breastfeeding prior to oral administration of RV vaccines, since no difference in vaccine efficacy has been demonstrated between breastfed and non-breastfed infants.^41^, ^42^, ^43^, ^44^

### Design of Experiments (DOE) Studies to Better Inform Formulation Development of RV3-BB Rotavirus Vaccine Candidate

The inactivation of the RV3-BB vaccine during storage and administration depends on a combination of factors including the inherent stability of the virus, the formulation used to surround the virus, and the effect of stress conditions (e.g., elevated temperatures, acidic pH, and agitation). The commonly used one-factor-at-a-time (OFAT) experimental design approach is valuable for scientific understanding of individual factors (e.g., type of excipient used or stress encountered) on RV3-BB stability by varying one parameter at a time. This approach offers easy design and data analysis, but is time-consuming and resource intensive in nature, especially with live virus vaccines where vaccine stability is monitored by viral titer assays. To accelerate formulation development efforts, a statistical experimental design approach, Design of Experiments (DOE) has become an important tool.^45^, ^46^, ^47^ DOE allows for more rapid screening of multiple experimental parameters by providing statistically significant multi-point solution (design space), along with information on interaction between input variables.^46^ Further, DOE is well suited for Quality-by-design (QbD) approach (as outlined in ICH Q8(R2)^48^) for assuring desired product quality across a wide design space as part of Chemistry, Manufacturing and Control (CMC) sections of regulatory filings.^49^ The use of DOE in formulation development of viral vaccine, however, is still in its early stages with a few recent reports including formulation development of stable OMV MenB vaccine candidate,^46^^,^^47^ and a combined empirical screening/DOE approach to identify stable lyophilized candidate formulations for a live Dengue vaccine candidate.^46^^,^^47^

In this work with candidate RV3-BB formulations, using DOE experiments with the sequential-addition *in vitro* digestion model to mimic oral delivery, the inter-relationships between various input variables (e.g., excipient concentrations and level of HCl addition) which affect RV3-BB titer losses and final solution pH were examined. The results demonstrated that a lower succinate concentration of ~200–350 mM is required (in presence of 5–8 mL Enfamil®) using 4 mL HCl addition or ~110–215 mM succinate using 3 mL HCl addition (vs. 400 mM succinate required to maintain solution pH in the forced degradation low pH model in the absence of Enfamil®). Considering addition of 3 mL HCl is likely representative of the infant gastric environment, 110–215 mM succinate should be sufficient to protect RV3-BB during oral delivery. This lower level of succinate in candidate formulations improves long-term storage stability of the RV3-BB vaccine compared to candidate formulations with 400 mM succinate (Kumar, Shukla et al., manuscript submitted).^20^

## Conclusions

In this work, we present two *in vitro* gastric digestion models to evaluate RV3-BB viral titer losses under conditions that mimic *in vivo* oral delivery. First, a forced-degradation model was used for screening and rank ordering the buffering capacity of RV3-BB stabilizers by comparing with Mylanta®. This model led to the selection of succinate, acetate and adipate as excipients providing sufficient ANC for protecting RV3-BB viral titers upon acid challenge without compromising storage stability. Second, a sequential-addition model was utilized to examine RV3-BB stability under conditions more representative of oral administration to infants including the effect of prefeeding. Based on DOE experiments combined with the sequential-addition model, 110–215 mM succinate was predicted to be optimal for oral administration of RV3-BB vaccine in pre-fed infants. These results were useful for designing new candidate RV3-BB oral formulations that could be benchmarked against preneutralization of gastric acid with Mylanta as has been done in RV3-BB clinical trials. These two *in vitro* gastric digestions models will hopefully be employed towards development of other orally administered vaccine formulations in the future.
